# Protected Areas Are Important for the Conservation of *Disa engleriana*, an Edible Orchid in Malawi

**DOI:** 10.1002/ece3.71778

**Published:** 2025-07-22

**Authors:** Blessings Tionge Chingagwe, Gift Gladson Moyo, Elizabeth Mwafongo, Tiwonge I. Mzumara, Jean Cossi Ganglo

**Affiliations:** ^1^ Earth Sciences Department Malawi University of Science and Technology Limbe Malawi; ^2^ Department of Biological Sciences Malawi University of Science and Technology Limbe Malawi; ^3^ National Herbarium & Botanic Gardens of Malawi Zomba Malawi; ^4^ Faculty of Agricultural Sciences University of Abomey‐Calavi Calavi Benin

**Keywords:** Bioclim, climate change, conservation, habitat, Orchidoideae, propagation

## Abstract

Edible orchids are increasingly threatened by unsustainable use in their natural habitats. Several studies highlight the need for propagation to counter this threat. However, a critical gap persists in understanding the environmental conditions that support these species in Malawi. This study aimed to identify potential habitats suitable for propagating edible orchids, focusing on *Disa engleriana* Kraenzl, 1893, to enhance ex situ conservation efforts. Present and future environmental data were sourced from WorldClim. Species occurrence data were obtained during field surveys and existing datasets. Using MaxEnt, continuous habitat suitability for *D. engleriana* was modeled based on presence‐only occurrence data, Bioclim variables, and elevation. The integration of ArcGIS allowed for a detailed analysis, reclassifying the continuous suitability map into suitable and unsuitable habitats. The results of the study show that most suitable habitats align with the boundaries of protected areas, emphasizing their critical importance in conservation planning. The study further found that suitable habitats for *D. engleriana* are typically at altitudes between 1500 and 1600 m, with temperatures not exceeding 15°C during the wettest months. In addition to that, the results revealed the negative impact of climate change on habitat suitability, projecting a decrease in suitable areas over the next 50 years.

## Graphical abstract

The article highlights that suitable habitats for *Disa engleriana* in Malawi alighn with the boundaries of protected areas emphasizing their importance in conservation. However, the species habitat is at risk due to climate change evidenced by the model projections. Therefore, propagating the species will help to conserve it in the wild while meeting the local economic demands.

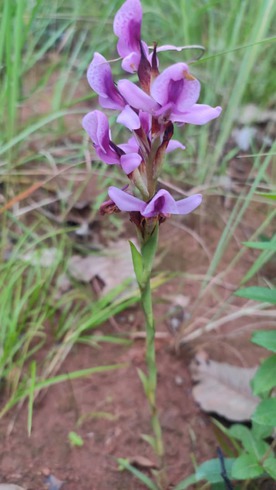

## 1 Background

Orchids represent the largest plant family with over 35,000 species (Schuiteman 2016; Besi et al. 2020). Orchids have significant economic value, serving as food, medicine, and decoration. Edible orchids, predominantly terrestrial, occur in upland and montane grasslands, comprising over 80 species from genera *Disa* Bergius, 1767; *Satyrium* Swartz, 1800; *Habenaria* Willdenow, 1805; and *Brachycorythis* Lindley, 1838 (Lalika et al. 2013). In Tanzania, Malawi, and Zambia, their tubers are important for food and livelihood. Their eye‐catching colors also provide esthetic value in Southern Africa (Kasulo et al. 2009; Lalika et al. 2013). However, increasing demand has led to overharvesting, putting these species at risk (Lalika et al. 2013).

**FIGURE 1:**
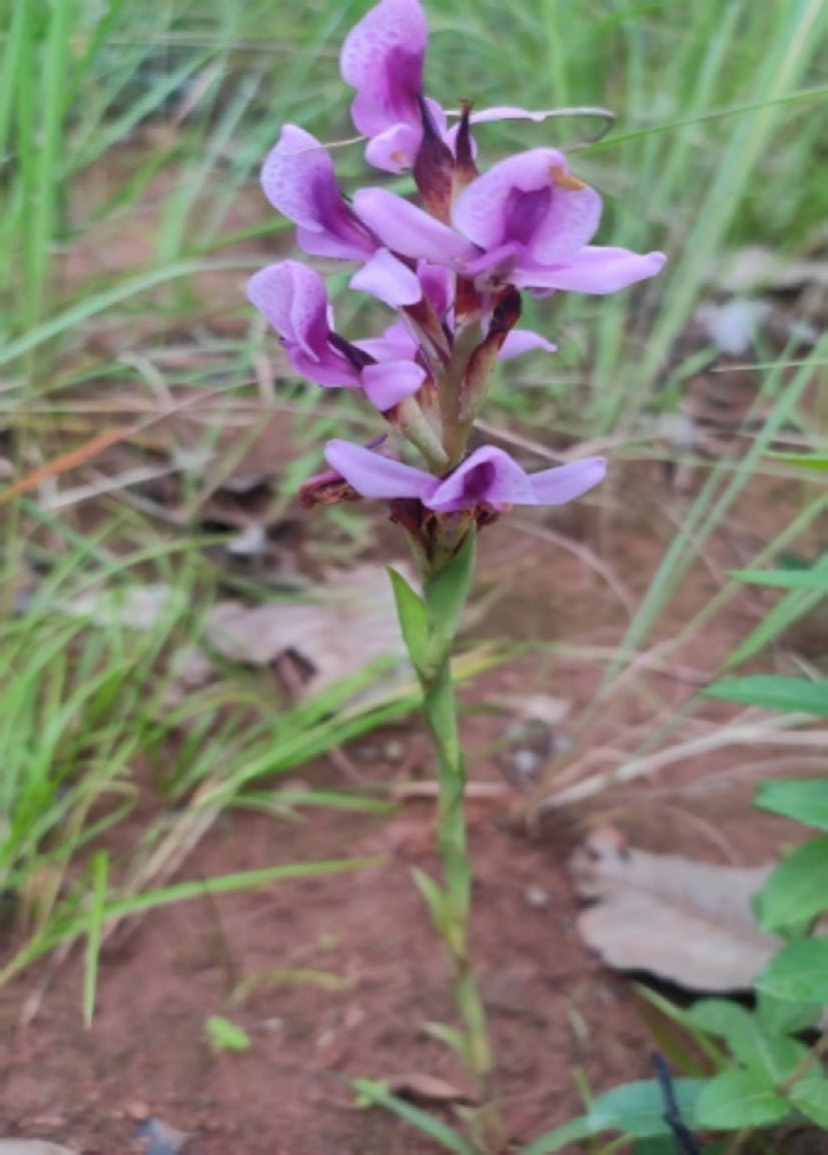
Photo of *Disa engleriana* in Nyika National Park. Photo Credit: B. Chingagwe.

Malawi alone hosts 420 orchid species, with 283 found in Nyika National Park (Lee 2020). Not only are the most common edible orchids in Malawi come from genera *Disa, Habenaria* and *Satyrium*, but *Disa engleriana* which is widely spread in the northern region of Malawi but also occurs in the bordering countries Tanzania and Zambia and is used to make Chikande/Chinaka, a traditional snack (Kasulo et al. 2009; Mapunda 2007). *D. engleriana*, characterized by its purple flowers with sterile shoots and two linear leaves (Figure 1), often blotched at the base of the plant (Kasulo et al. 2009), is known to be widely spread across the northern region. The increasing commercial demands for orchid tubers, coupled with inadequate regulation, have exacerbated pressure on the wild populations. Unfortunately, edible orchids face the threat of extinction in the wild due to escalating food demand and trade, leading to overexploitation and unsustainable harvesting (Dombo et al. 2002; Kasulo et al. 2009; Lalika et al. 2013). Invasive alien species (IAS), habitat destruction, fragmentation, and degradation pose further threats to orchid populations (Liu et al. 2015). The study by Kasulo et al. (2009) shows that *D. engleriana*, along with other orchid species like *Satyrium cursonii*, *Satyrium buchananii* and *Satyrium amblyosaccos*, is becoming scarce while demand is increasing. The scarcity of these orchids has led to escalated cross‐border trading, particularly between Zambia and Malawi, raising concerns about their sustainability in the wild (Kasulo et al. 2009). Despite these threats, conservation actions for edible orchids remain limited. Presently, there are no clearly documented conservation actions directly targeting *D. engleriana*. However, the species benefits from habitat conservation actions within protected areas. Therefore, protected areas remain the main refuge for *D. engleriana*, yet enforcement and community engagement are minimal. Orchid diversity has been documented by the National Herbarium & Botanic Gardens of Malawi, while propagation attempts are still in their initial stage (Mwanyambo 2003).

Several species distribution modeling approaches have been widely used to model species distribution to provide evidence‐based management and conservation decisions, and it has proven to have a rigorous predictive power in both bigger and smaller data records (Bariotakis et al. 2019). Ecological niche modeling (ENM) is used to understand the environmental conditions suitable for species distribution (Ouyang et al. 2022; Peterson 2003). ENM combines species occurrence data with bioclimatic factors to predict suitable habitats, considering the impact of climate change (Wani et al. 2021). Maximum entropy (MaxEnt), a statistical tool within ENM, uses occurrence data to generate habitat suitability models (Bariotakis et al. 2019). These models can help identify regions for orchid propagation, addressing both sustainability and the livelihoods of local communities (Peterson 2003). The models provide a science‐based foundation for targeted conservation actions that can enhance societal action (Guisan et al. 2013). Areas identified as suitable habitat could guide pilot *D. angleriana* cultivation for local communities, while unsuitable areas would be ideal for supporting other livelihood activites, hence, reduced pressure on the *D. angleriana* in the wild.

Several studies have recommended propagation as a potential solution to increasing demand for orchid tubers (Bariotakis et al. 2019; Challe and Struik 2008; Kasulo et al. 2009; Wani et al. 2021; Zambrano and Bennett 2002). Cultivating these orchid species not only ensures sustainability but also contributes to improving the livelihoods of local communities and meeting cross‐border market demand. However, it seems inadequate initiatives have been undertaken to propagate the orchids in other geographical spaces. Moreover, scarcity of information entails that little or no study has been conducted to reveal the required information on the comparable environmental conditions suitable for propagation of orchids in ex‐ situ. In view of this, there is great need of conducting further research into the environmental conditions for orchid propagation, especially given the growing threats from climate change (Wraith 2020). The information gathered through this study would be directly in line with sustainable development goals (SDGs) 13 and 15, focusing on climate action, biodiversity conservation, and sustainable harvesting practices, aligning with Malawi's Vision 2063 for environmental sustainability (Clémençon 2021; United Nation 2018; National Planning Commission 2020).

This study therefore seeks to understand optimal environmental conditions necessary for ex situ cultivation of *D. engleriana* by (i) mapping the potential habitat suitability for the species, (ii) projecting the future species niche over a period of 50 years in the mapped habitat, and (iii) assessing the impact of climate change on the distribution of the species over a period of 50 years.

## 2 Materials and Methods

### 2.1 Study Area and Sampling Design

**FIGURE 2:**
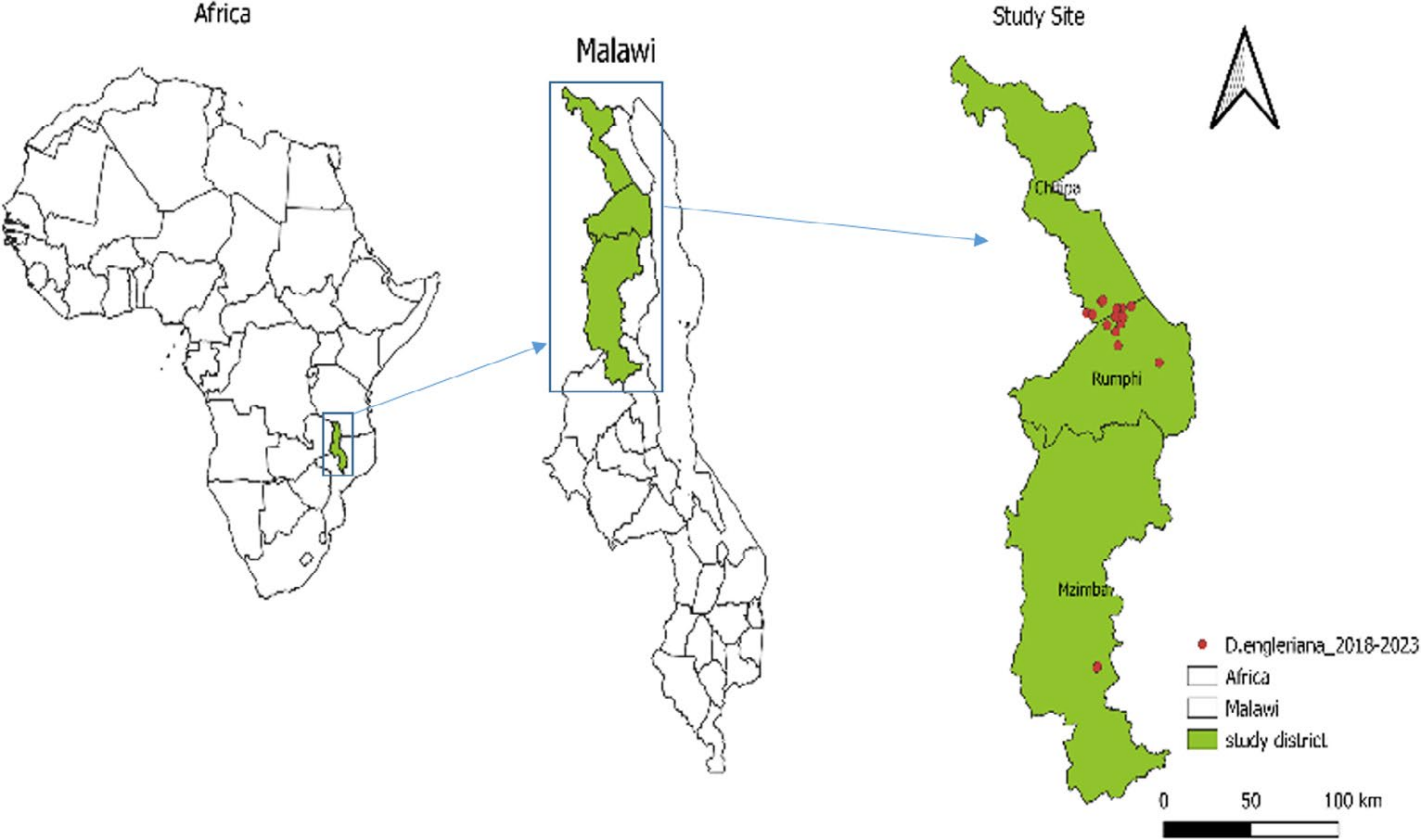
The three districts in northern Malawi as the study site.

The study focused on the districts of Mzimba, Rumphi, and Chitipa (Figure 2) in northern Malawi (Hijmans 2023) which were purposively selected due to the high prevalence of unsustainable harvesting and cross‐border trade of edible orchids in the region. Previous studies (Namoto and Draft 2018) in northern Malawi have provided occurrence data utilized in this research, and a prior study explored local perceptions of orchid cultivation in the region (Lee 2020).

Mzimba is the largest district in Malawi and covers an area of 10,430 km^2^ (Mzimba District 2022) with an estimated population of 870,000. It has a warm tropical climate, receiving annual rainfall between 650 and 1300 mm (Gama et al. 2014), and its average temperatures range from 10°C to 29°C (World Weather online 2025; Climate top 2024) depending on the time of the day/night and part of the year. Rumphi district is bordered by Chitipa to the north, Karonga to the northeast, Mzimba to the south, and Nkhata Bay to the southeast. Rumphi is a predominantly agricultural district, experiencing diverse climatic conditions, with temperatures ranging from 11°C to 30°C and annual rainfall between 900 and 1200 mm (Chinsinga 2008). Chitipa spans an area of 4288 km^2^ and has a population of 234,927 (NSO 2019). According to World Weather (2023), the district experiences temperatures between 11°C and 33°C, with annual rainfall reaching up to 1300 mm. The district is bordered by Karonga and Rumphi and shares an international boundary with Tanzania and Zambia (Firm 2012) (World Weather 2023). Generally, it shows that these districts under study have similar climatic conditions.

Nyika National Park (Rumphi/Chitipa districts), Lunyangwa Valley (Mzimba), Lusangazi Forest (Mzimba), Mtangatanga Forest Reserve (Mzimba), and Luwawa Forests (Mzimba) were surveyed for species occurrence, as these locations were initially identified by the model as potential habitats for *Disa engleriana*. Nearly all accessible sites were visited, including those with an AUC (area under curve) below the threshold of 0.7, following the guidelines set by Mzumara et al. (2019). Notably, only Nyika National Park and Mafinga Hills met the AUC threshold of > 0.7.

A total of 30 sampling points were identified using a prototype model which was developed in MaxEnt using *Disa engleriana* species data, and areas with AUC of > 0.7 were targeted for sampling. The 30 sampling points were selected to balance data coverage with the logistical constraints of time and resources. Sampling was conducted in March, coinciding with the flowering period of *Disa engleriana* (Mapunda 2007) to maximize the likelihood of locating the species. At each sampling point, 10 quadrants measuring 5 m by 5 m were laid along a 50‐m transect. This method was chosen to ensure a systematic and comprehensive assessment of the species' presence. The use of multiple quadrants enhances the reliability of the data by accounting for spatial variability within each site (Elzinga et al. 2001), ensuring data representativeness across the broader habitat and improving the robustness of the study results (Price 2014).

### 2.2 Data Collection and Analysis

#### 2.2.1 Bioclimatic and Species Data

The WorldClim v2.1 dataset providing averaged climate data from 1970 to 2000 at a 30‐arc‐second resolution (~1 km at the equator) with 19 bioclimatic variables was sourced from Fick and Hijmans (2017). Future climate projections were obtained using the Model for Interdisciplinary Research on Climate, Earth System version 2 for Long‐term simulations (MIROC‐ES2L) model under the ssp126 scenario from the Coupled Model Intercomparison Project Phase 6 (CMIP6), covering 2061–2080. MIROC‐ES2L was selected for its comprehensive representation of soil nutrient impacts and carbon–nitrogen interactions (Hajima et al. 2019), enhancing accuracy in ecosystem simulation despite potential biases requiring cautious interpretation.

The initial species occurrence dataset comprised of data from various platforms: seven records from the Global Biodiversity Information Forum (GBIF); five records from the National Herbarium and Botanical Gardens (NHBG); and eight records from the Forest Research Institute of Malawi (FRIM). Field surveys provided 14 records of the *D. engleriana* occurrence data. Recent data dating back to 2018 were used, while older records were excluded due to potential obsolescence as a result of potential significant land use changes and possible georeferencing inaccuracy.

The species data cleaning was done using OpenRefine version 3.5.1 to eliminate duplicates and empty entries. The software was also used for extensive exploration utilizing clustering and faceting methods. The resultant dataset with 22 entries was subsequently transferred to ArcGIS for visualization, pinpointing and eliminating of outliers. This refined dataset is accessible on GBIF (Mzumara‐Gawa et al. 2024). Considering MaxEnt's ability to perform well even with smaller sample sizes (*n* < 30) in comparison to other models (Gaikwad et al. 2011), the study confidently utilized the resultant dataset of 22 occurrence points (*n* = 22).

### 2.3 Modeling Present and Future Habitat Suitability

MaxEnt 3.4.4 was used to model the current potential distribution of *Disa engleriana*, employing environmental predictor variables from 1976 to 2000. To identify the predictor variables contributing most significantly to the model, the MaxEnt algorithm was run five times, systematically eliminating variables with less than 1% contribution each time.

Presence‐background method using MaxEnt version 3.4.4 was employed to predict the future potential suitable habitat of species. MIROC‐ES2L models for 2061–2080 corresponding to current variables were used as future predictor variables. The data were divided into 75% for training and 25% for testing (Kohavi and Edu 1993). The model was run five times with cross‐validation and 500 iterations per run to ensure enough convergence time. A jack‐knife test was performed to assess the significance of each predictor variable, and clamping confined the algorithm to the training data range. Attributes from MaxEnt, programmatically defined and verified across various species and environmental factors including the quantity of species records and sample selection bias extent (Smith et al. 2012), were utilized. The algorithm produced a model to understand the current habitat suitability and forecast a 50‐year scenario of the suitable habitat. ArcGIS was used to convert MaxEnt outputs into binary data, distinguishing suitable from unsuitable habitats based on minimum training presence (3.8%) and maximum training sensitivity plus specificity (31%). The present and future minimum and maximum models were compared, and the change in suitable habitat area over time was calculated in ArcGIS using the pixels of estimated size of 1 × 1 m^2^.

### 2.4 Model Evaluation

The model's performance was evaluated by calculating the mean AUC from five replications. The study used discrimination capacity (Sillero et al. 2021) as measured by area under curve (AUC) of the receiver operating characteristics (ROC) plot to evaluate the performance of the model. The model with an AUC between 0.5 and 0.7 was classified as poor model performance, 0.7–0.9 was classified as good performance, and greater than 0.9 was classified as high performance (Mzumara et al. 2019).

### 2.5 Possible Study Limitations

*Inaccessibility of Sampling Sites*: Data collection for orchids, which typically flower during the rainy season, was conducted during this period, increasing the likelihood of encountering inaccessible sites. Accurate fieldwork timing required careful consideration of weather forecasts, but some locations remained unreachable. This uneven sampling could introduce bias, potentially leading to overemphasis on more extensively sampled areas. To address this issue, a presence‐only approach was employed in habitat suitability modeling, minimizing the risk of inaccuracies associated with pseudo‐absence data.

## 3 Results

### 3.1 Environmental Data for Model Development

Table 1 shows variable percent contribution and corresponding permutation importance percent (%) contribution of each environmental variable used in the determination of habitat suitability. Results show that mean temperature of wettest quarter (bio8) registered the highest contribution to the model (40.6%), followed by elevation (38.3%) and isothermality (bio3) being the lowest (4.4%).

**TABLE 1:** Percentage contribution and permutation importance of variables: Percentage contribution is the relative contribution of each variable to the model. Permutation importance measures the decrease in training AUC by permuting the values of the variable across training points.

Variable	Percent contribution	Permutation importance
Mean temperature of wettest quarter (bio8)	40.6	0
Elevation	38.3	64.7
Annual mean temperature (bio1)	8.7	33.2
Min. temperature of coldest month (bio6)	8	2.2
Isothermality (P2/P7)*(100) (bio3)	4.4	0

### 3.2 Verified *Disa engleriana* Abundance and Distribution in Selected Areas

*Disa engleriana* data were scarce from all sources which were explored including its occurrence in the wild making it challenging to obtain a sample size of 30 points. Despite numerous studies on edible orchids being conducted and Kasulo et al. (2009) reported that *D. engleriana* is said to be widespread across northern Malawi, data were still scarce. However, according to Gaikwad et al. (2011) MaxEnt performs well even with smaller sample sizes, that is, *n < 30* as compared to other models; hence, modeling of the species suitable habitat proceeded, and the output model was satisfactory.

**FIGURE 3:**
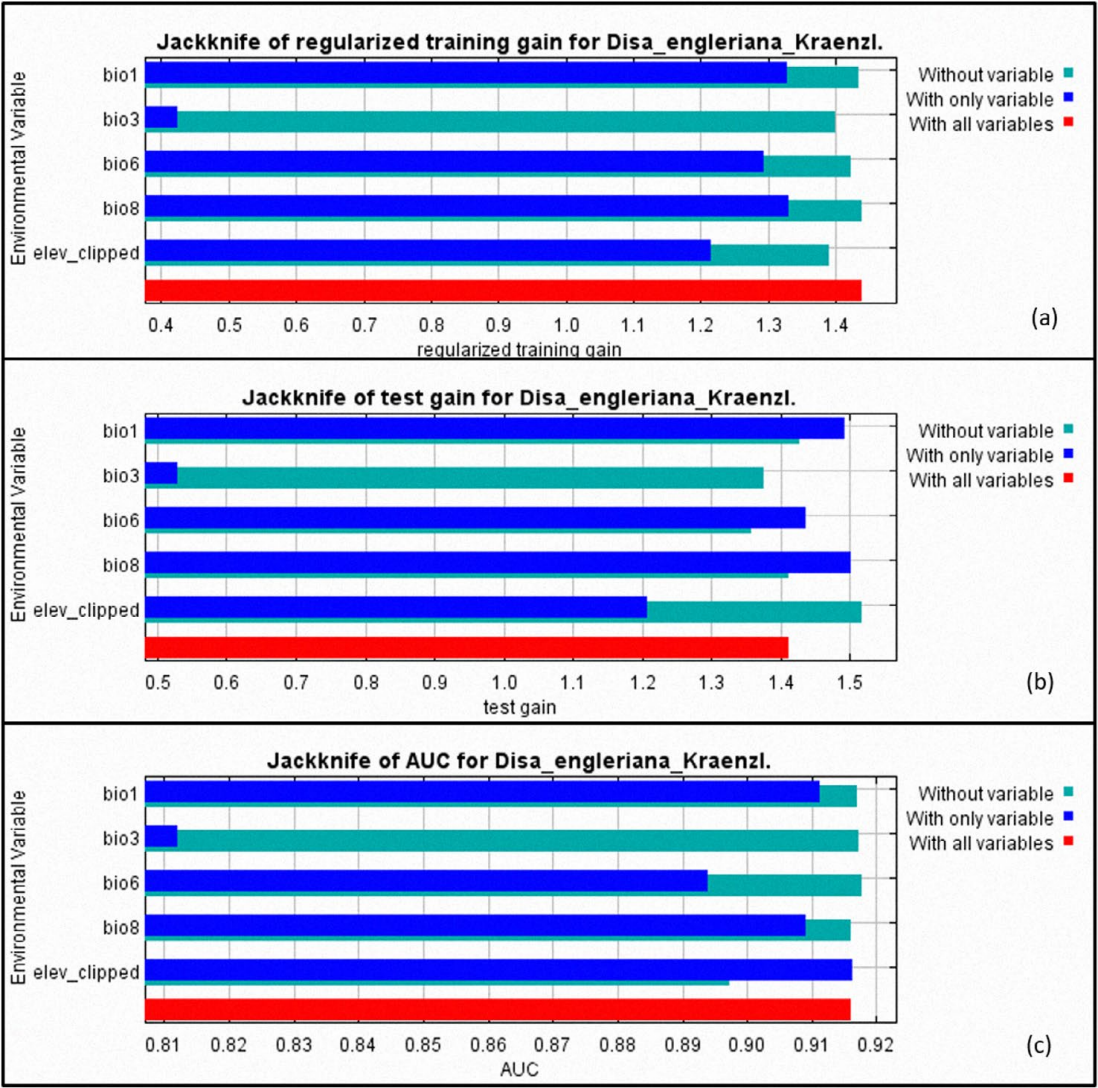
Jackknife test of variable importance where (a) is against regularized training gain, (b) is against test gain, and (c) is against area under curve (AUC). Dark blue indicates the variable importance to the model if it is used independently. Light blue indicates variable importance when it is not included in the model.

According to the jackknife test results (Figure 3), the annual mean temperature (bio1) and mean temperature of wettest quarter (bio8) had the highest training gain, hence high influence on the AUC. Isothermality (P2/P7)*(100) (bio3) had the least useful information because its contribution rate to the model was very low when used independently.

### 3.3 Model Validation

**FIGURE 4:**
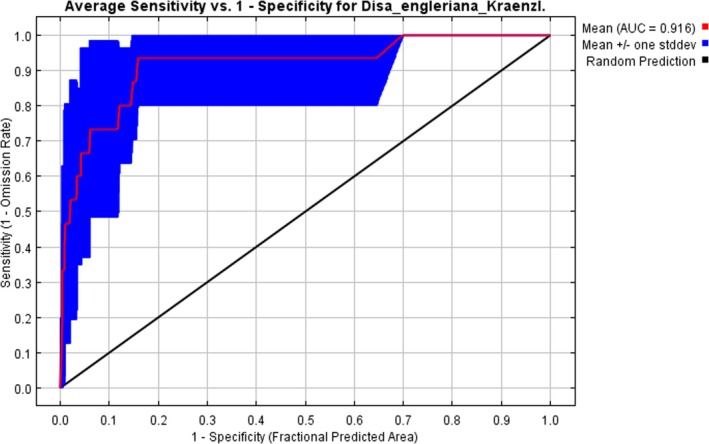
Receiver operating characteristics (ROC) curve for *Disa engleriana* model performance. The black diagonal line represents random prediction where AUC is 0.5 and is a baseline for comparison. The red line represents the mean performance of the model with AUC of 0.916 which indicates high model performance. The blue‐shaded area shows the variation in model performance across different replicated cross‐validations, being represented by mean +/− one standard deviation.

On Figure 4, the ROC curve illustrates the predictive performance of the species distribution model for *D. engleriana*, showing sensitivity (true positives rate) against 1‐specificity (false positive rate). The model calibrated at a 10% training presence threshold in order to account for any uncertainties in the occurrence data produced an average AUC of 0.916, indicating strong model performance compared to random predictions (shown in black in Figure 4), with a low standard deviation of 0.002, signifying minimal variation in AUC values. The red axis in Figure 4, representing the model's performance, remains above the random prediction (black diagonal axis), further demonstrating the model's consistency and reliability.

**FIGURE 5:**
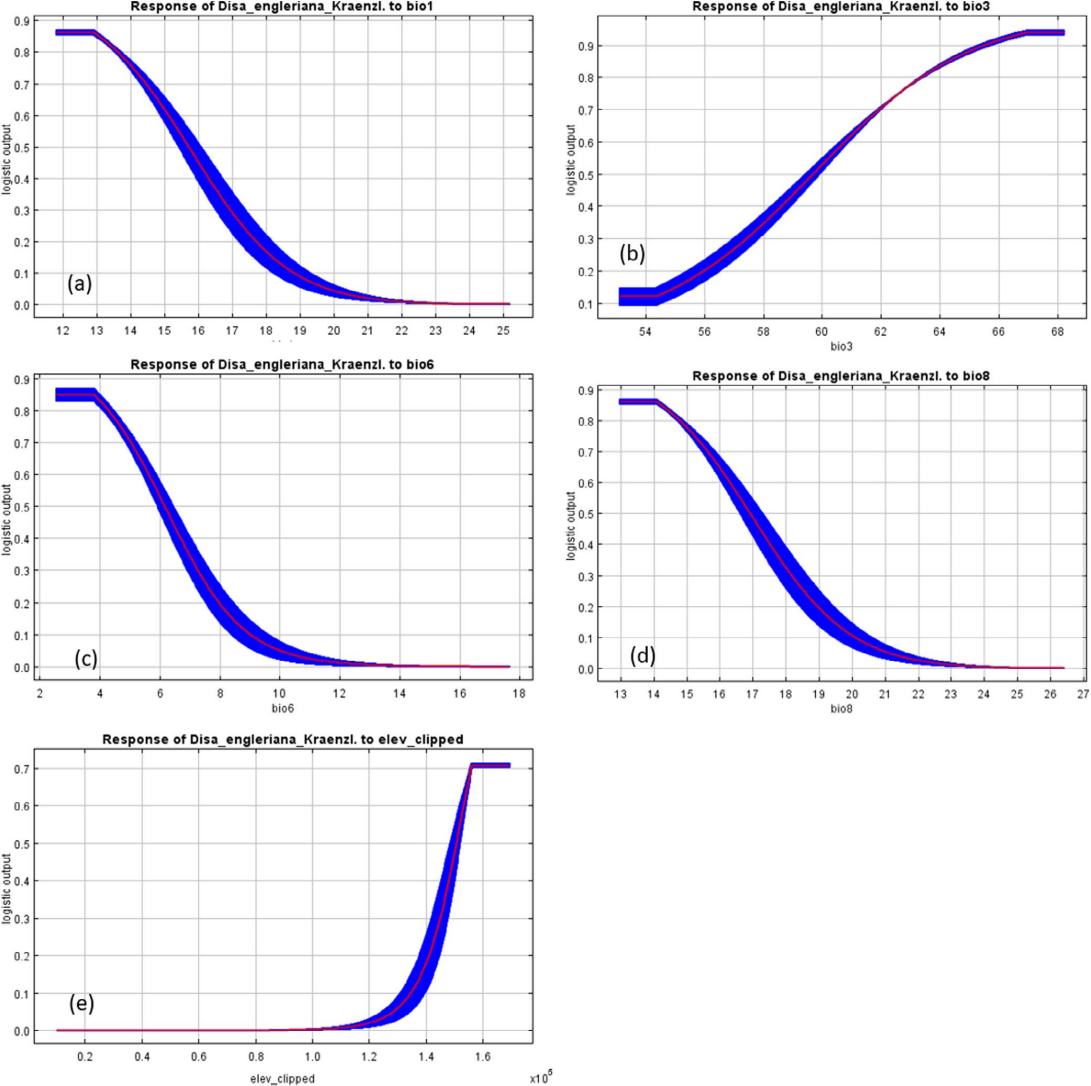
Response curves for the key environmental variables: (a) annual mean temperature, (b) isothermality (P2/P7)*(100), (c) mean temperature of wettest quarter, (d) min. temperature of coldest month, (e) elevation.

According to the variable response curves (Figure 5), favorable conditions would be: (i) annual mean temperature (bio1) of around 11.8^o^C and 15^o^C and that higher temperature reduces the probability of species habitat suitability; (ii) isothermality (monthly diurnal temperature variability‐bio3) of between 54.5% and 66.9%; (iii) mean temperature of wettest quarter (bio8) of between 13°C and 14°C, but higher temperatures would not support the species habitat suitability; (iv) minimum temperature of coldest month (bio6) of between 2.9°C and 3.9°C; and (v) elevation of between 1500 and 1600 m above sea level is favorable for the species habitat suitability while lower elevation does not support the suitability of the habitat for the species.

### 3.4 Habitat Suitability

**FIGURE 6:**
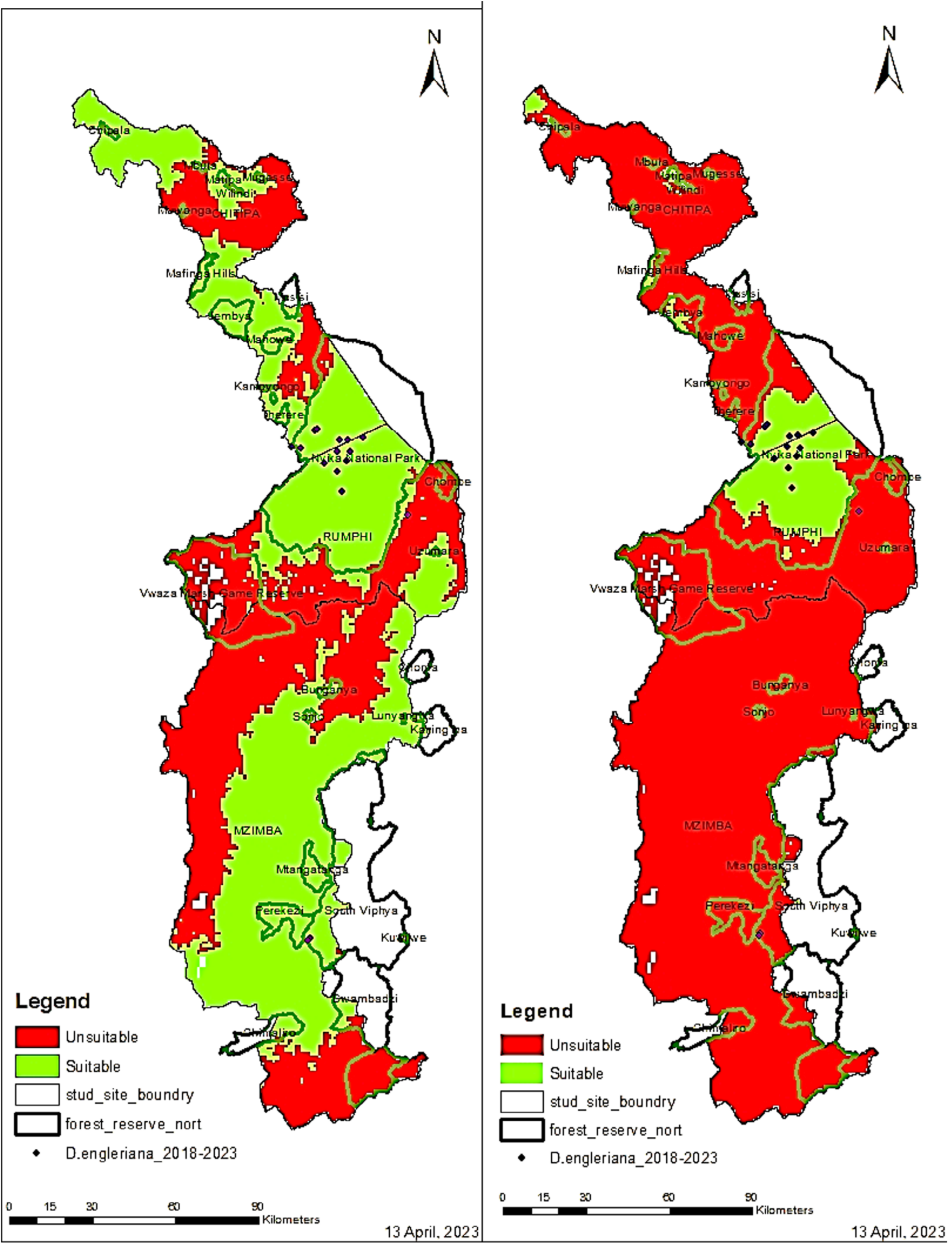
Present Habitat suitability models for *Disa engleriana*. Areas in green represent suitable habitats while areas in red are deemed unsuitable. Green outlines indicate forest reserve boundaries and black outlines represent study site boundaries. Black dots represent observed occurrences of *D. engleriana* from 2018 to 2023 period. Protected areas are an important part of the models as they show the overlap between the conservation areas and the suitable habitats. MaxEnt was used to produce the models based on version 2.1 bioclimatic variables: Annual mean temperature (bio1), mean temperature of wettest quarter (bio8), isothermality (bio3), minimum temperature of coldest month (bio6) and elevation. (a) Represents habitat suitability model at a minimum training presence of 0.038 and (b) shows that suitable habitat has significantly decreased at a maximum training sensitivity plus specificity of 0.313, as indicated by larger proportions of areas in red.

The potential current habitat suitability model (Figure 6) was calibrated using a binary classification in ArcGIS where (a) represents habitat suitability of a logistic threshold at a minimum training presence of 3.8% with a statistical significance of 0.3072 while (b) represents the habitat suitability of a logistic threshold at a maximum training sensitivity plus specificity of 31% with a statistical significance of 0.0036. At present, the results show that suitable habitats based on the maximum threshold are concentrated in Nyika National Park, Mafinga Hills, and Chipala Hills, all of which are designated protected areas. Nyika National Park and Mafinga Hills being transboundary sites, straddling between Malawi and Zambia, foster collaborative management of the species through resource sharing and coordinated policies. At a minimum threshold, the suitable habitat seems to spread across all the three districts even outside gazetted protected areas.

### 3.5 Projected Species Habitats

The pixels' count was used to compute the area of the suitable and unsuitable areas (Table 2). In the present projections, at minimum threshold (3.8%), 1081.73 km^2^ is predicted to be suitable for the species compared to 786.04 km^2^ unsuitable habitat; while at a maximum threshold (31%), 504.05 km^2^ is predicted to be suitable habitat and 4802.89 km^2^ unsuitable for the species. Future projections show a decline in suitable habitat to 32.4% at minimum threshold and 0.59% at maximum threshold, alongside a notable expansion of unsuitable area from 5306.94 km^2^ to 8239.3 km^2^, particularly under maximum threshold.

**TABLE 2:** Habitat suitability classified by area for present and future projections.

	Present projections				Future projections			
	Minimum training presence		Maximum training sensitivity and specificity		Minimum training presence		Maximum training sensitivity and specificity	
Habitat status	Area (Km^2^)	Percentage (%)	Area (Km^2^)	Percentage (%)	Area (Km^2^)	Percentage (%)	Area (Km^2^)	Percentage (%)
Suitable	1081.73	57.9	504.05	9.5	673.65	32.4	48.38	0.59
Unsuitable	786.04	42.1	4802.89	90.5	1402.34	67.6	8190.92	99.41
Total	1867.77	100	5306.94	100	2075.99	100	8239.3	100

### 3.6 Impact of Climate Change on the Distribution of *Disa engleriana*

**FIGURE 7:**
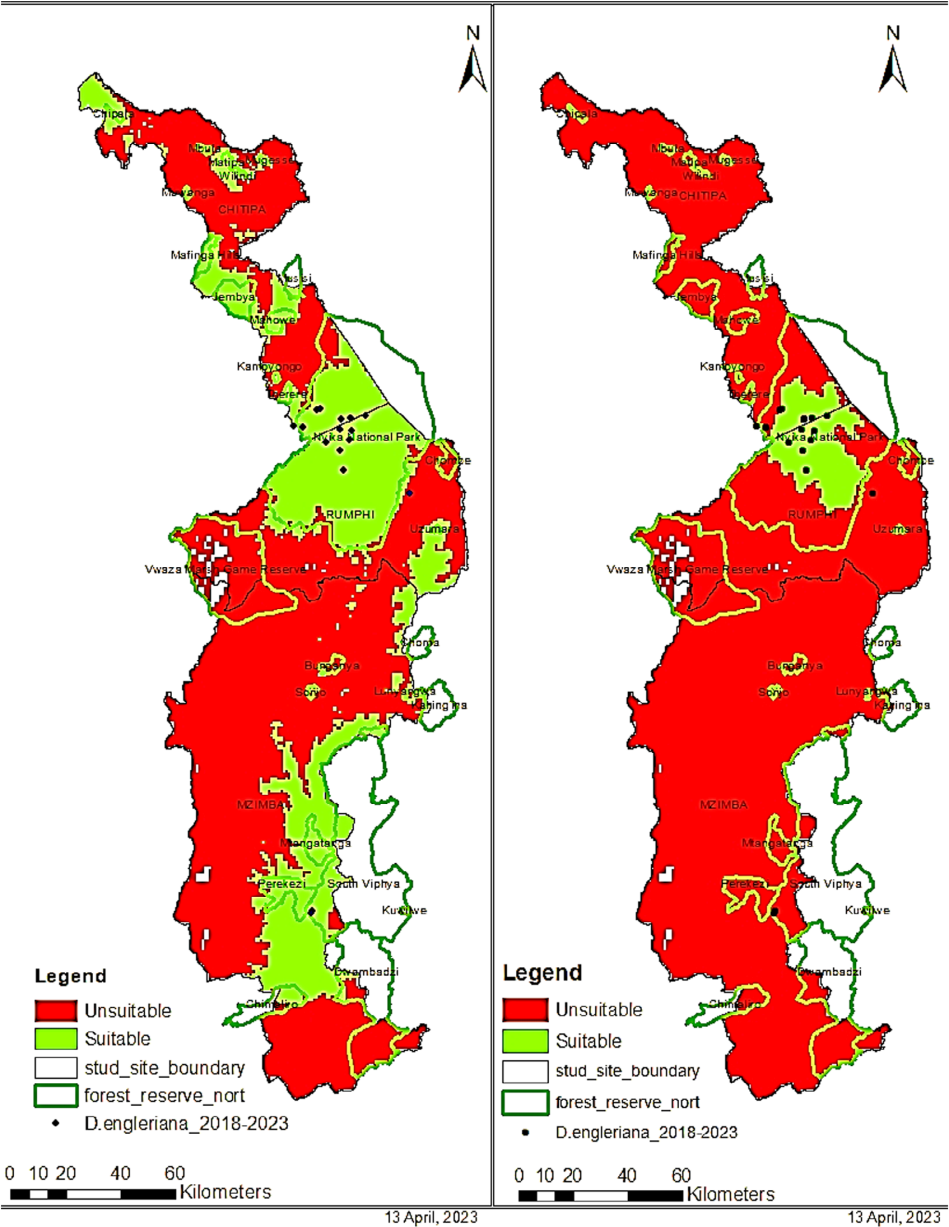
Future habitat suitability models for *Disa engleriana*. Areas in green represent suitable habitats while areas in red are deemed unsuitable. Green outlines indicate forest reserve boundaries and black outlines represent study site boundaries. Black dots represent observed occurrences of *D. engleriana* from 2018 to 2023 period. Annual mean temperature (bio1), mean temperature of wettest quarter (bio8), isothermality (bio3), and minimum temperature of coldest month (bio6) were derived from MIROC‐ES2L models from 2061 to 2080 to predict a 50‐year climate effect on the species. (a) Represents habitat suitability model at a minimum training presence of 0.038 and (b) shows that suitable habitat has significantly decreased at a maximum training sensitivity plus specificity of 0.313, as indicated by larger proportions of areas in red.

Climatic conditions change with time, and a model was developed to project the effects of the bioclimatic conditions in the next 50 years (Figure 7). The predicted suitable habitat was compared with the current suitable habitat to compute the difference in area change using the same minimum and maximum thresholds as in the current model. Future projections based on climatic variation at a minimum threshold of 673.65 km^2^ are estimated as suitable while at a maximum threshold of 48.38 km^2^ is predicted to be suitable. The potential suitable area is predicted to reduce by 25.5%, which is significant for a statistical significance of 0.307 and 8.91%, which is insignificant for a statistical significance of 0.004 for minimum and maximum thresholds, respectively.

## 4 Discussion

### 4.1 Environmental Data

Among the 19 bioclimatic variables analyzed, four significantly contributed to the model, with mean temperature of wettest quarter (bio8) (Table 1) having the most substantial impact. Consistent with Jodamu (2001), the genus *Disa* thrives in cool habitats with temperatures ranging from 10°C to 26°C, tolerating up to 30°C with sufficient moisture and airflow. Nyika National Park supports high orchid diversity, including *Disa engleriana*, has annual rainfall of between 1000 and 2200 mm (Namoto and Draft R. 2018) and annual temperatures ranging from 0°C to 21°C. Recent data (Malawi Government 2022) show Mzimba, Rumphi, and Chitipa districts receiving average annual rainfall of 900, 650, and 2000 mm, respectively, between 1991 and 2020. The model identified suitable habitats at 1500–1600 m elevations, within *D. engleriana*'s natural range from 1000 to 2000 m (Royal Botanical Gardens Kew 1995). The model's predictions for annual mean temperature (bio1), minimum temperature of coldest month (bio6), and mean temperature of wettest quarter (bio8) align with observed temperatures in Mzimba (10°C–29°C), Rumphi (12°C–30°C), and Chitipa (9°C–29°C) (Ventures 2018). Although other factors like soil and vegetation type could influence *D. engleriana*'s occurrence, this study highlights elevation, rainfall, and temperature as the primary determinants. In addition to soil and associated vegetation, orchid mycorrhizal fungal distribution and abundance are critical for natural recruitment of seedlings and establishment of orchid colonies (McCormick et al. 2004), emphasizing that fungal photosynthetic orchids can easily be propagated.

### 4.2 Present and Projected Habitat Suitability

*Disa engleriana* typically thrives in Miombo woodlands and associated grasslands (Mapunda 2007), a habitat accurately identified by the model. Analysis using ArcGIS revealed that occurrence points for *D. engleriana* were predominantly within Nyika National Park, while suitable habitats extended across Chitipa, Rumphi, and Mzimba districts. This distribution aligns with Kasulo et al. (2009), suggesting that many potential habitats are within protected areas, which remain relatively undisturbed. However, potential habitats outside protected areas were also identified, particularly when using minimal training presence thresholds for both current and future models. Field verification showed that *D. engleriana* was scarce outside Nyika National Park, possibly due to overexploitation, habitat degradation, or limited study in those areas. In addition, the concentration of the species in Nyika National Park may also be due to the species being substantially protected in Nyika National Park. The other interpretation may be that the species may have limitations for wider dispersal. Therefore, managers of protected areas may consider developing conservation guidelines to enhance in situ conservation with a focus on monitoring *D. engleriana* populations, prevent illegal harvesting, and restore degraded habitats. Additionally, engaging bordering communities for an integrated community‐based conservation initiative to promote alternative livelihoods and sustainable harvesting is crucial.

The estimated area of occupancy for *Disa engleriana* varies significantly based on the threshold used. With a minimum threshold, the area was approximately 1081.73 km^2^, whereas at a maximum threshold, it was reduced to 504.05 km^2^, reflecting a 31.79% decrease. MaxEnt models generally predict a larger optimal habitat than the actual occupied areas (Dai et al. 2022), which is useful for identifying potential conservation areas. However, MaxEnt's data‐driven approach may be less precise for future projections compared to process‐based models that track biological processes over time (Smith et al. 2012). Therefore, this study's predictions may be limited by data constraints.

### 4.3 Impact of Climate Change on the Distribution of *Disa engleriana*

The model predicted a significant reduction in species suitable habitat as a result of climate change over a period of 50 years. At maximum threshold, suitable habitat for *Disa engleriana* is seen to be confined to Nyika National Park, while all other sites become unsuitable. However, at the minimum training presence threshold, both present and future projections show that suitable habitat would still be prevalent outside Nyika National Park, aiding future conservation and possible cultivation of the species. The projected 2°C temperature increase over the next 50 years (Ganglo 2023) poses significant challenges for *D. engleriana* conservation. Such warming driven by global greenhouse gas emissions is likely to exacerbate habitat loss for the species. This climate‐driven shift underscores the urgency for proactive measures.

MaxEnt, which has successfully predicted species habitat suitability even with limited data, was used in this study (Dai et al. 2022). Ganglo et al. (2017) recommended MaxEnt for its consistency in model transfers, and it produced robust results here. However, MaxEnt assumes a species prevalence of 0.5, meaning the model predicts that species occur in half of the potential sites, which is not always accurate (BCCVL 2021). After analyzing 19 rainfall, temperature, and environmental factors, five were found to significantly contribute to the model. Temperature‐related factors and elevation were prioritized for their strong influence on species distribution, suggesting they have the most impact on the geographic range of *Disa engleriana*. It is important to note that other local factors, such as climate variability, may also affect species distribution. The study recognizes that orchids are highly dependent on specific fungal associations for germination and growth (McCormick et al. 2004). As such, further research is recommended to determine areas most suitable for ex situ conservation considering soil conditions and mycorrhizal fungi.

## 5 Conclusion and Recommendations

The models indicate that protected areas remain the most suitable for the species, with areas bordering these regions showing high potential for propagation. However, orchid poaching in protected areas poses a significant threat, while populations outside these areas are nearly nonexistent. To circumvent this issue, artificial propagation via in vitro methods must be done to plant orchids outside the protected areas to both avoid people entering the reserves and collecting orchids from these areas. Incentives to plant orchids in large scale outside the protected areas must be the priority. This will help develop large colonies for sustainable utilization.

Climate change is expected to negatively impact the species' suitable habitat, with projections indicating that, by 2080, suitable areas will be restricted to Nyika National Park at the maximum threshold. This significant reduction underscores a direct correlation between climate change and habitat suitability for *Disa engleriana*. Briefly, annual mean temperature (bio1), isothermality (bio3), minimum temperature of the coldest month (bio6), mean temperature of the wettest quarter (bio8), and elevation are crucial environmental factors for the thriving of *D. engleriana* in ex situ conditions. The study highlights a direct correlation between bioclimatic conditions and habitat suitability, with optimal conditions for *D. engleriana* being elevations between 1500 and 1600 m and temperatures not exceeding 15°C during the wettest months.

The findings indicate that future predictions, which do not account for overexploitation and habitat loss, foresee a significant reduction in suitable habitat for *Disa engleriana*, suggesting a risk of further resource depletion. This study offers valuable environmental insights for the species' propagation, emphasizing that while protected areas serve as key conservation sites, the most suitable locations for cultivation are areas bordering the reserves. Since local communities around these areas are primary harvesters, targeting them for ex situ conservation efforts is recommended. Given that this study is not exhaustive, we advocate for propagation trials in these identified sites to validate the findings. We also recommend use of more data to feed into the model to validate the effectiveness of the model with our findings.

Policy recommendations include enhancing in situ conservation within the designated protected areas to safeguard the species' natural habitat. Additionally, propagating *D. engleriana* in botanical gardens is advised due to the species' rapid decline in the wild. Lastly, a concerted effort to gather comprehensive occurrence data for *D. engleriana* is essential to expand the data pool for future research and informed decision‐making.

## Author Contributions

**Blessings Tionge Chingagwe:** conceptualization (lead), data curation (lead), formal analysis (lead), funding acquisition (lead), investigation (lead), methodology (lead), project administration (lead), resources (lead), writing – original draft (lead). **Gift Gladson Moyo:** project administration (equal), supervision (lead), writing – review and editing (lead). **Elizabeth Mwafongo:** supervision (supporting), validation (equal), writing – review and editing (equal). **Tiwonge I. Mzumara:** conceptualization (equal), methodology (supporting), project administration (supporting), supervision (supporting), validation (equal), writing – review and editing (supporting). **Jean Cossi Ganglo:** formal analysis (equal), methodology (equal), validation (equal).

## Conflicts of Interest

The authors declare no conflicts of interest.

## Data Availability

The species occurrence data underpinning this study is available at Global Biodiversity Information Facility (https://www.gbif.org/dataset/3c97e43d‐e670‐4c17‐9cbe‐5403cd245063).
